# Arrhythmias During Pregnancy: Ventricular Tachycardia in the Setting of Maternal Beta Thalassemia Minor

**DOI:** 10.7759/cureus.64599

**Published:** 2024-07-15

**Authors:** David M Langley, Roger C Kelly, Marshall A Frank

**Affiliations:** 1 Emergency Medicine, Florida State University College of Medicine, Sarasota, USA; 2 Emergency Medicine and EMS (Emergency Medical Services) Medicine, Florida State University College of Medicine, Sarasota, USA

**Keywords:** structural heart disease, arrhythmias, β-thalassemia, pregnancy, non-sustained ventricular tachycardia

## Abstract

Non-sustained ventricular tachycardia (NSVT) poses significant risks during pregnancy, particularly in patients with underlying conditions such as β-thalassemia. We present a case of a 29-year-old pregnant woman with a history of β-thalassemia minor who experienced NSVT at 27 weeks gestation. Despite initial concerns for structural heart disease, the workup was unrevealing. Challenges in medication selection and risk assessment were addressed in the context of maternal and fetal well-being. This case underscores the importance of a multidisciplinary approach involving cardiology, obstetrics, and hematology in managing NSVT during pregnancy, emphasizing risk stratification, collaborative decision-making, and long-term follow-up to ensure optimal outcomes for both mother and fetus.

## Introduction

Non-sustained ventricular tachycardia (NSVT) is a self-limiting arrhythmia consisting of at least three consecutive ventricular beats lasting less than 30 seconds. This arrhythmia can be produced by direct mechanical, electrical, or chemical stimulation of the myocardium and is often noted during acute coronary syndromes, myocarditis, hypoxia, and electrolyte abnormalities, particularly hypokalemia. Management is directed at correcting the underlying illness [1]. Of most concern to the emergency physician, NSVT is considered a warning sign for the development of sustained ventricular tachycardia (VT) and can potentially lead to sudden cardiac death (SCD) [2]. We present a case of a pregnant 29-year-old female with a history of β‐thalassemia minor found to have NSVT. The thalassemias are a group of hereditary disorders caused by defective hemoglobin synthesis and have varied complications. The management of NSVT is addressed in the context of this underlying condition in the following case report.

## Case presentation

A 29-year-old gravida one para zero, at 27 weeks gestation, with a history of β‐thalassemia minor presented to a freestanding emergency department (ED) with complaints of palpitations, pre-syncope, and an intermittent sensation of a racing heart over the preceding four days. Her symptoms were exacerbated when standing, particularly while working (she was employed as a hairdresser), and alleviated upon sitting. She reported a past medical history of anemia managed with iron supplementation, and her medications included a daily prenatal multivitamin. She denied taking other medications or supplements, tobacco, drug or alcohol use. She denied other past medical or surgical histories. Initial examination revealed mild sinus tachycardia at a rate of 110/minute, and a gravid uterus without tenderness. A thorough evaluation was initiated to explore the etiology of her symptoms.

**Figure 1:**
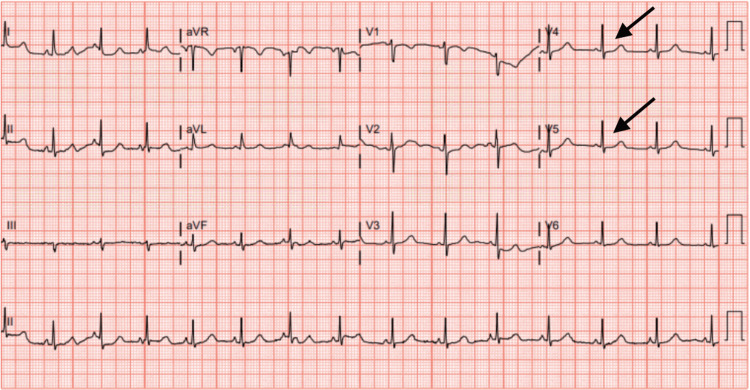
Initial 12-lead electrocardiogram obtained in the emergency department with non-specific ST segment depressions most noticeable in leads V4-V5, as highlighted by the black arrows

**Figure 2:**
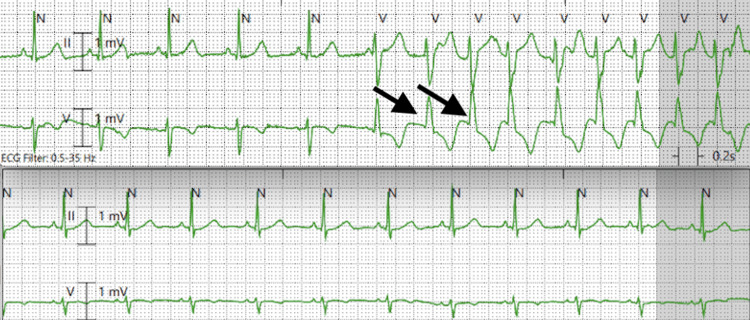
Telemetry strip demonstrating a wide complex tachycardia as highlighted by the black arrows (top) with resumption of normal sinus rhythm on subsequent recording (bottom)

Laboratory investigations in the ED revealed anemia (hemoglobin of 9.5 grams/deciliter), albeit above baseline, which the patient reported to be around 9 grams/deciliter, and a thyroid profile within normal limits. Notably B-type natriuretic peptide (BNP) was 70 picograms/milliliter, and timed lab draws for troponin levels taken two hours apart were 7 and 4 nanograms/liter, all well within the normal range. Potassium, magnesium, and calcium levels were 3.5 millimole/liter, 2.1 milligrams/deciliter, and 8.9 milligrams/deciliter, respectively. Computed tomography angiography (CTA) of the thorax revealed posterior-dependent atelectasis but showed no evidence of pulmonary embolism. An EKG taken on arrival showed nonspecific ST segment depressions (Figure 1) most notable in leads V4 and V5. She was about to be discharged when the patient was observed to have a wide complex tachycardia at a rate of approximately 150/minute captured on telemetry (Figure 2, top), spontaneously converting to normal sinus rhythm on subsequent telemetry recordings (Figure 2, bottom). A decision was made to transport her to a tertiary care center for further evaluation due to concern for NSVT. 

Cardiology initiated metoprolol 12.5 mg three times daily, with recommendations to replace potassium and magnesium levels with a goal of greater than 4 millimoles/liter and 2 millimoles/liter, respectively. Obstetrics followed along closely during admission. Inpatient evaluation included transthoracic echocardiogram (TTE) and exercised-induced stress testing. TTE demonstrated an ejection fraction (EF) of 60-65%, no wall motion abnormalities, and no significant valvular pathology. The stress test revealed no signs of ischemia, and the patient demonstrated excellent functional capacity with no associated symptoms. She remained in sinus rhythm during testing. On hospital day 2, she developed mild hypokalemia (3.4 millimoles/liter), and potassium was replaced per institutional protocol. She demonstrated borderline hypotension the same day, with the lowest documented blood pressure recorded as 82/60. She reported lightheadedness at this time. After one hour of crystalloid fluid resuscitation (one liter of lactated Ringer's solution), the blood pressure was recorded as 111/66. Based on these findings, cardiology postulated that her previous episode of NSVT was triggered by volume depletion and hypokalemia, although no arrhythmias were captured during this admission. Inpatient cardiac magnetic resonance imaging to evaluate for possible infiltrative cardiomyopathy was considered but ultimately deferred due to the potential for harm to the fetus with exposure to gadolinium contrast. The patient was ultimately cleared for discharge with a Zoll Arrhythmia Management System (AMS) patch (ZOLL Medical Corporation, Chelmsford, Massachusetts, US) for continuous cardiac monitoring with instructions to follow up with cardiology, hematology, and obstetrics within two weeks of discharge. She was instructed to have a repeat complete blood cell count and metabolic panel drawn for follow-up and to continue daily oral iron supplementation. After discharge, she was seen by hematology, and due to her persistent anemia with continued intermittent episodes of presyncope and palpitations, she underwent two iron transfusions. Concomitantly, the patient was seen by cardiology; oral electrolyte supplementation was continued and monitoring devices were changed from the Zoll AMS to a Holter monitor. This device revealed runs of sinus tachycardia during symptomatic episodes. She was subsequently cleared by cardiology, as no further electrolyte derangements or arrhythmias were noted during this period of observation. Hematology transitioned the patient to oral iron supplementation after laboratory studies performed at 35 and 36 weeks gestation revealed improvement in hemoglobin at 10.3 and 10.4 grams/deciliter, respectively. Her clinical course thereafter was uncomplicated, and a healthy female child was delivered by spontaneous vaginal delivery at 40 weeks and 4 days.

## Discussion

According to Forget et al., thalassemias are a group of inherited disorders caused by defective synthesis of globin chains, resulting in an inability to produce normal adult hemoglobin [3]. The principal effect of these disorders is microcytic, hypochromic, and hemolytic anemia. These disorders are most common in those of Mediterranean, Middle Eastern, African, and Southeast Asian descent and are thought by Sankaran et al. to be protective against malaria [4]. Β‐thalassemia is inherited in an autosomal recessive pattern and leads to a decrease or even absence of normal β‐hemoglobin side chain production, leading to, in some instances, severe anemia refractory to transfusion therapy. The long-term complications for those homozygous for the β-globin mutation can be severe and are well-documented, though rare, in patients with β-thalassemia minor. Patients with β-thalassemia minor are heterozygous for the β-globin mutation and have only mild microcytic anemia [5-6]. Hemosiderosis and iron overload in various tissues lead to inevitable endocrinopathies, hepatopathies, arthropathies, and perhaps pertinent to our patient, cardiomyopathies. It must be said, however, that unless another aggravating condition is present that affects iron metabolism and contributes to iron overload, according to Ladis et al. the likelihood of an underlying cardiomyopathy in a patient with β-thalassemia minor is small [7].

Despite this rarity, structural heart disease was considered a potential culprit in this young patient with no prior cardiac history and newly diagnosed arrhythmia. If structural heart disease was indeed confirmed to be present, serious consideration should be given to using an ICD. Challenges arise in the selection of medications commonly utilized to treat arrhythmias to minimize potential harm to the fetus. Traditional anti-arrhythmic drugs, such as beta-blockers, may be used, whereas second-line drugs, such as amiodarone, are used for treatment-resistant cases while considering the side-effect profile [8]. Every attempt was made during hospitalization to identify an underlying trigger, though the impact of thalassemia as a potential culprit may have been underestimated. There are well-documented cardiac complications of thalassemia, as well as the complications of the treatments of this disease, heart failure, arrhythmias, pericarditis, and pulmonary hypertension. In these advanced disease states, disease heart transplant is considered definitive therapy [9].

Pertinent to this case, however, it must be noted that intermittent episodes of VT have been observed in patients with β-thalassemia; in one study, it was stated that ventricular arrhythmias are more specific for iron cardiotoxicity, and the presence of couplets and NSVT should raise clinical suspicion thereof [10]. Unfortunately, Bayar et al. did not delineate the difference in rates of arrhythmias among the β-thalassemia major and minor patients but did show that patients who developed sustained arrhythmias were often refractory to conventional antiarrhythmic therapy and required placement of an ICD [11]. One recent study suggested a high incidence of SCD among young patients without clinical evidence of cardiac disease, with a 27% occurrence rate over a 26-year observation period [12]. Moreover, according to Taher et al., this risk extends beyond the more severe form to any patient with thalassemia and results in increased morbidity as the patient advances in age [13]. Further studies are certainly warranted to explore the unique considerations in managing ventricular arrhythmias during pregnancy, particularly in populations heterozygous for β-thalassemia.

## Conclusions

This case highlights the challenges in managing NSVT in a pregnant patient and the increased risk for subsequent sustained ventricular tachyarrhythmias and SCD in those patients diagnosed with β‐thalassemia minor. The important task of the ED physician is to facilitate risk stratification of patients and initiate the collaborative efforts of cardiology and obstetrics. As our case has shown, hematology consultation should also be strongly considered. Clearance for discharge with continuous monitoring and long-term follow-up with consulting services should be arranged prior to discharge to ensure the well-being of both the mother and the developing fetus.
